# The role of artificial intelligence in colon polyps detection 

**Published:** 2020

**Authors:** Pezhman Rasouli, Arash Dooghaie Moghadam, Pegah Eslami, Morteza Aghajanpoor Pasha, Hamid Asadzadeh Aghdaei, Azim Mehrvar, Amir Nezami-Asl, Shahrokh Iravani, Amir Sadeghi, Mohammad Reza Zali

**Affiliations:** 1 *Department of Computer, West Tehran Branch, Islamic Azad University, Tehran, Iran*; 2 *Gastroenterology and Liver Diseases Research Center, Research Institute for Gastroenterology and Liver Diseases, Shahid Beheshti University of Medical Sciences, Tehran, Iran *; 3 *Gastroenterology and Hepatobiliary Research Center, AJA University of Medical Sciences, Tehran, Iran*; 4 *Basic and Molecular Epidemiology of Gastrointestinal Disorders Research Center, Research institute for Gastroenterology and Liver Diseases, Shahid Beheshti University of Medical Sciences, Tehran, Iran*; 5 *Research Center for Cancer Screening and Epidemiology, AJA University of Medical Sciences, Tehran, Iran *

**Keywords:** Artificial intelligence, Deep learning, Polyp detection, Image processing, Computer-assisted, Colonoscopy.

## Abstract

Over the past few decades, artificial intelligence (AI) has evolved dramatically and is believed to have a significant impact on all aspects of technology and daily life. The use of AI in the healthcare system has been rapidly growing, owing to the large amount of data. Various methods of AI including machine learning, deep learning and convolutional neural network (CNN) have been used in diagnostic imaging, which have helped physicians in the accurate diagnosis of diseases and determination of appropriate treatment for them. Using and collecting a huge number of digital images and medical records has led to the creation of big data over a time period. Currently, considerations regarding the diagnosis of various presentations in all endoscopic procedures and imaging findings are solely handled by endoscopists. Moreover, AI has shown to be highly effective in the field of gastroenterology in terms of diagnosis, prognosis, and image processing. Herein, this review aimed to discuss different aspects of AI use for early detection and treatment of gastroenterology diseases.

## Introduction

 Based on the reports from the Iranian Annual National Cancer Registration, colorectal cancer (CRC) is the fifth and third major cause of death in women and men, respectively (1). It is well-known that almost all cases of CRC originate from colon polyps (2). The majority of adenomatous polyps could progress to CRC within approximately ten years (3). Currently, the best effective method to prevent CRC is regular screening and early polyp detection (4). Colonoscopy is the most reliable tool for polyp detection and resection (4). Despite significant capabilities of colonoscopy in screening and polyp detection, specific polyps could be missed during the procedure and thus may result in the process of cancer development (5, 6). Similar biases are frequently observed in other diagnostic modalities such as CT colonography (7). With the recent advances in computer technology using artificial intelligence (AI), numerous studies have been carried out to assess the utility of AI in medical image processing such as radiology and gastroenterology. The application of AI in medical imaging analysis has been investigated in several medical fields such as radiology, neurology, orthopaedics, pathology, ophthalmology, and gastroenterology (8). AI has also achieved noticeable results by using specific methods such as machine learning (deep learning) in the field of image processing in medicine (9). AI has been consistently implemented in the medical field in various methods such as machine learning, decision trees, and artificial neural networks (ANNs) (10, 11). Using and collecting a huge number of digital images and medical records has led to the creation of big data over a time period. Given the rapid advances of AI in recent years, gastroenterologists and physicians need to learn AI tools as well as its strengths and weaknesses. Physicians should also be able to use AI in real clinical practice in the near future. As a result, machine learning could greatly impact medical decision making due to an increasing need for the interpretation of large amounts of clinical data. In this study, the aim was to discuss the most important aspects of AI use in the gastroenterology field. 

**Computer-aided polyp detection and diagnosis**

We still do not have a full understanding of how the brain works. As long as human beings continue to rely principally on visual inspection to detect endoscopic images, we will never dominate the previous obstacles of individuality causing wider variability in observations (12). Computers appear to be more efficient than the human eye and brain in terms of image processing and detection (13). With the exception of the human brain, an independent and realistic operator has an advantage which can be used by any doctors without any particular practice (14). In general, AI is regarded as an intelligent machine which is capable of learning and problem solving by itself (15). Currently, machine learning is the most common method used in AI (16). This method automatically builds mathematical algorithms based on input data (training data) that can predict and decide in uncertain conditions without human intervention (17). 

AI has been used in gastroenterology images for processing, diagnosis, prognosis, and analysis (18). Due to the importance currently given to big data, the collection of large-scale medical digital images provides grounds for making effective use of AI which provides essential resources for machine learning by itself (9). Furthermore, limitations of traditional machine learning methods can be overcome by increasing computing power with graphics processing units (10). This leads to an increased efficiency of AI, especially when using deep learning technology, and has proven to perform well in the analysis of medical images (16).

There are some possible methods for polyp detection and recognition. Machine learning algorithms can have considerable influence over precise interpretation of endoscopic images (14). Pilot trials on the use of a convolutional neural network have illustrated promising outcomes. (19, 20). In an ideal manner, neural networks should be nurtured by the most visual score (finally based on advanced imaging methods) provided by an overall central reading (21).

**Figure 1:**
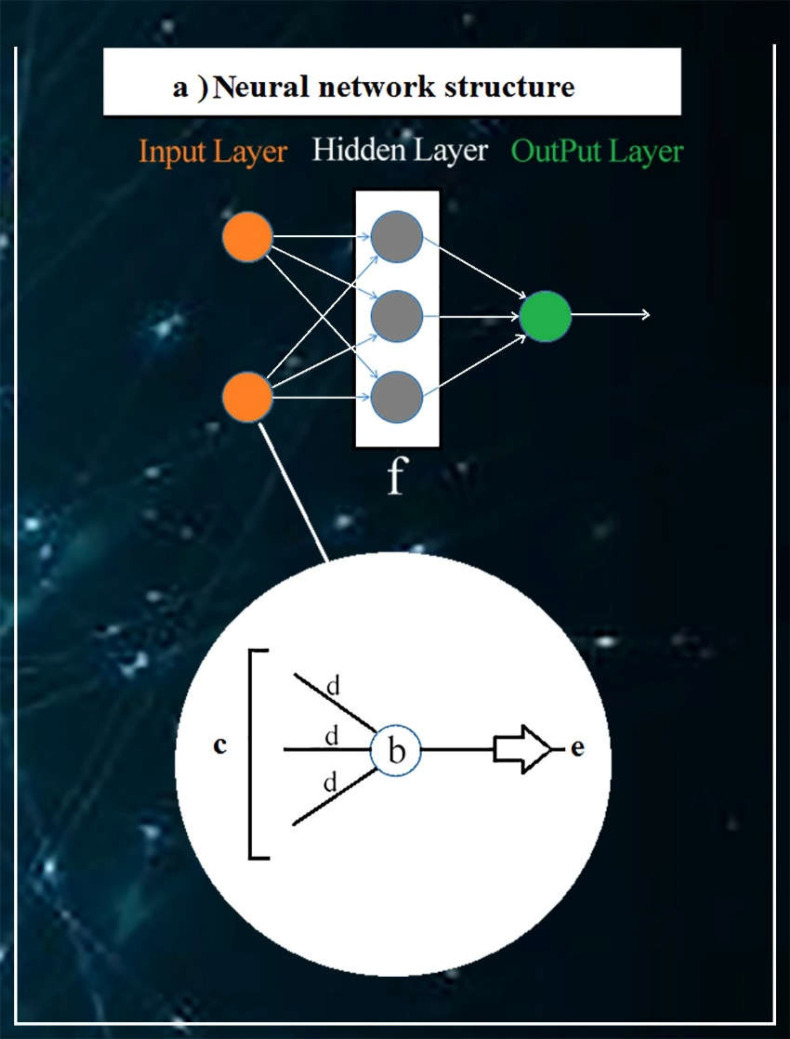
**a)** **N** **eural network structure: **An Artificial Neural Network (ANN) is a computational pattern to process information, resembling the way biological neural systems in the human brain process data and information. ANNs have been applied to speech recognition, image analysis, and adaptive control **b)** **Neuron: **Each pattern of a neural network is made up of numerous neurons often called nodes. A node, as the primary computation unit, is a mathematical function that simulates the functioning of a biological neuron. A neuron consists of a set of inputs, a set of weights, and an activation function. The neuron transforms input data into a single output, which can then be used as input for another layer of neurons. **c) Input: **Data imported from the outside world to the neural network are called inputs. Inputs are modified and summed by the weight vectors. The “Input Layer” does not perform any computation (input nodes) and just moves data to hidden layers. **d) Weight: **Each neuron consists of a weight vector, which is equal to the number of inputs to that neuron. Then, given that each input raw data can have many features, the amount of weight is determined by those features. For instance, for a prediction model of diabetes, weights are height, age, smoking, and so on.** e) Output:** The output neurons result from hidden layers. *************dear writer, please CHECK the previous sentence**************Then, they are planned for transferring computational information from the neural network to the outside world. Therefore, the domain of the output is controlled by an activation function in order to reach an acceptable range of output, which is often between 0 and 1. Also, the output of the neuron can then be used as input to the neurons of another layer, which could repeat the same computation. **f) Hidden nodes: **The hidden layer is made up of numerous neurons, which are called hidden neurons. They perform a mathematic function and computation process to transform input data and information to signals, to be transferred from the input layer to the output layer

CNN is one of the seven kinds of artificial neural networks (Figure 1), including CNN, recursive neural network, recurrent neural network, multilayer perceptron, long short-term memory, sequence-to-sequence models, and shallow neural networks. Despite the fact that disease symptoms are considered to be crucial in diagnostic justification, there are no reliable techniques to confirm this information as a diagnostic tool (22). To solve non-linearity inherent in the relationship between disease symptoms and underlying pathology, ANNs are used as highly-adaptive modeling mechanisms (61). Some novel algorithms are represented by ANNs as the solution for non-linear problems that are highly complex for conventional statistical analysis such as CNNs (23). 

To date, the application of image processing with AI in deep learning through CNNs has significantly increased in medical fields for diagnostic imaging. Moreover, automatic detection of polyps (and cancer diagnosis) in colonoscopic images is affected by CNNs (24). CNNs are particularly applied to analyze visual imagery. They also have applications in image and video recognition, skin cancer classification, diagnostic and radiation oncology and diabetic retinopathy, histologic classification of gastric biopsy, and characterization of colorectal lesions using endocytoscopy (24, 25). As a result, medical image detection of gastrointestinal cancer at an early stage by CNNs is the single most effective measure which can lead to a reduced rate of gastrointestinal cancer mortality (57).

Image processing could be performed by extracting pixel data from high quality images and integrated detection patterns in the algorithm (i.e., computer aided diagnosis algorithm based on neural networks) (14). These non-discriminatory image data can be further linked to histological data. Moreover, it has been recently shown that this approach is more compatible with ulcerative colitis histology and is considered to be the best predictor of sustained clinical remission in these patients (26).

Furthermore, the use of big data provides convenient input data for training. Moreover, the rapid development of computational power allows researchers to overcome previous restrictions (27). Input images are filtered using multiple special filters to extract specific features and create multiple feature plans. This processing method for filtering is called convolution (28). A learning method is important for the convolution filter to create the best feature plans for the success of CNNs (21). 

Given that AI is currently concentrated on the gastrointestinal field in image analysis, several machine learning samples have demonstrated promising outcomes in disease diagnosis and prediction (29). Although, endoscopic imaging programs have reduced mortality caused by gastrointestinal diseases, these diseases still lead to death on a large scale globally, which is a huge burden economically (10) Hence, gastroenterologists have become enthusiastically interested in the application of AI and its analysis, especially the use of CNN and support vector machines in medical image processing. In addition, AI has been significantly used in the diagnosis of non-digestive diseases as well as infections (28, 30). 

Although there has been promising progress in polyp auto-identification methods, they have not led to a prospective evaluation (31-33). However, in a study performed by Klare et al. (34) new validation software was established in real-time conditions (performed on 55 sequences of daily colonoscopies). Similar results were reported in terms of detection capability between doctors and the developed software. Accordingly, polyp diagnosis and adenoma diagnosis rates reported by endoscopists were 56.4% and 30.9%, respectively. However, these rates were reported as 50.9% and 29.1% by the software, respectively. In another study, Wang et al. (35) implemented a new deep learning algorithm using data from 1,290 patients, which was then validated with 27,113 new colonoscopy images collected from 1,138 patients. The

**Figure 2:**
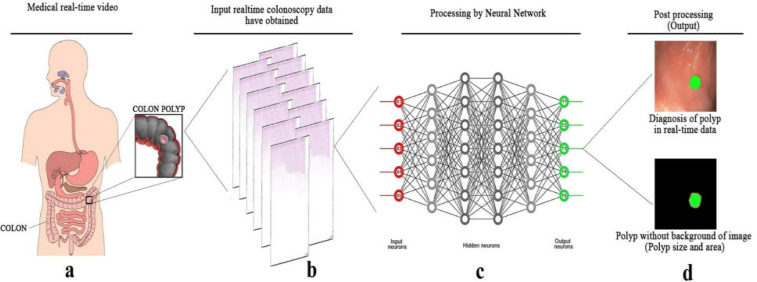
The neural network of artificial intelligence in real time data processing. This schematic outlines a neural network method for a representative detection task, such as diagnosis of colon polyp. **a)** The first stage of image processing by neural network is taking colon real-time videos of patients visited by doctors to diagnose polyps. **b)** Real-time videos will be prepared for a functional method, in particular properties of images. As a result, these provided data called input data. **c)** In this stage, a neural network uses input data for crucial processing, which contains plenty of neuron layers in its method. Following that, promising outcomes in polyp detection are reached as output data with two types of processes. **d)** The provided output illustrates two kinds of data which are separated into a marked polyp in real-time video and polyp with no background of tissue

proposed technique indicated significant efficiency with a sensitivity of 94.38% and the area under receiver operating characteristic (AUROC) of 0.984 in at least one polyp diagnosis (35). In the different areas of gastrointestinal fields, development of an AI technique using colonoscopy has shown promising results in polyp detection, which is frequently observed during the colonoscopy procedure (36). 

AI training is defined as a collection of numerous medical images including real-time video and static images that are the primary source of image processing, resulting in statistical outputs for further interpretation (Figure 2). These data could be used as a valuable source for detection of missing colorectal polyps that are directly linked to the development of colon cancer (10). Urban et al. (37) benefited from a CNN system to detect colonic polyps. Moreover, they used 8,641 hand-labeled medical images and 20 colonoscopy videos in several combinations as well as training data (37). The CNN technique in polyp detection with real time yielded an AUROC of 0.991 and accuracy of 96.4%. Moreover, it contributed to the distinction of nine additional polyps in comparison to the ones detected by expert endoscopists in the application of test colonoscopy videos (28). 

**Computer-assisted magnetic resonance imaging (MRI) in colon**

The currently available clinical tools for assessing the colon (e.g. constipation by radiography) have not shown a reliable diagnostic performance, as detected by observers (38). In several studies, automated and semi-automated methods have been used for virtual colonography to segment colon images using computerized tomography (CT) scan (39-45). Binary thresholding, region growing, and morphological operations have been applied in most of these researches for colon segmentation from CT images with little or no user interaction (39-43, 45). The studies rely on gas filling of the colon or contrast enhancement to obtain adequate contrast of the colon and colon wall. This subsequently led to alternation of the colon morphology by distension (bloating) and unnecessary radiation (21). The study of the unready colon morphology to prepare and quantify colonic regional volumes without ionizing radiation has been facilitated by MRI (46). Image segmentation must be performed manually in order to acquire the colon size, which is a tedious and time-consuming process. Image segmentation using semi-automated software can be beneficial to reduce segmentation time (47, 48). Furthermore, in terms of reliability of detection, automated methods have shown better results when compared to manual assessments, which may ultimately result in reducing the variability of observations (49). Noticeable reduction of the contrast between gut tissue and surrounding tissue compared to imaging using contrast agents or bowel preparation has led to complicated automatic segmentation in the MRI of unprepared colon (50). In this regard, results related to automatic colon segmentation of MRI images are restricted due to the presence of image in homogeneities (51). In the semi-automatic technique, the colon volume does not indicate fasted state or full state, but rather may lie in between (52). However, the purpose of the current study was to validate a semi-automated image segmentation method as the colon status does not affect observers’ agreement on segmentation (52).

MRI has been used to evaluate the effect of corticotrophin releasing hormone on regional volume of colon. Moreover, it can also be applied in drug studies to assess the effect of drugs on the regional size of colon and fecal distribution (53). A modern semi-automated method using unprepared colon and feces from MRIs has raised hopes for use in clinical practice in the near future (54). Quantification to colon volume in the unprepared colon rather than routine non-quantitative examinations such as radiography can be observed in this method. This method enables objective measures on fecal volume and colon morphology that could also increase diagnostic value, particularly in patients with constipation and abdominal pain (50). Thus, this method can be used to guide physicians towards more accurate diagnosis as well as efficient therapies (52).

**AI pitfalls in gastroenterology**

In this section, several disadvantages of AI related to its direct implication for computer-aided detection and diagnosis are discussed. These potential disadvantages of AI in colonoscopy were pointed out in a prospective study using real-time polyp characterization. It was shown that the time required for colonoscopy was raised from 35 to 47 seconds per polyp characterization (36). Moreover, polyp characterization may reduce endoscopists' concentration and could result in missing or misidentification of polyps (55). It should also be noted that reliance on AI alone could reduce the number of skillful endoscopists in the near future. Further prospective studies are needed to assess these drawbacks in addition to the effectiveness of AI (36). 

Although many studies have been conducted on polyp characterization systems, they have been evaluated and developed using video images and saved images. These images are often taken as ideal images of diagnosed lesions and polyps, as selected by endoscopists. Thus, they may appear to be ineffective in daily practice (56). Moreover, histopathological findings may not always exist. In this regard, lack of data on the severity of inflammation and dysplasia, as observed in ulcerative and Crohn’s colitis, is another restriction (19, 57). AI promises to empower physicians in reducing the significant burden of clinical documentation to improve the quality of patient care and enhance physician-patient interaction (58). For instance, AI could help radiologists validate raw image data to facilitate recognizing complex patterns, which could ultimately result in unbiased reports (59).

**AI future in gastroenterology**

To overcome human error in tumor detection and avoid excessive biases, the use of AI in the gastroenterology field appears to have a bright future ahead when compared to the use of advanced endoscopic techniques alone. Endoscopic methods have not shown promising results in assessing and diagnosing inflammatory bowel diseases (60). Despite numerous reports regarding the utility of AI tools, most studies conducted on this issue were retrospective in nature (61). In these circumstances, the presence of potential inherent bias in selecting data cannot be ignored (9). Hence, significant validation of AI performance is crucial before its application in real clinical practice (62). Over fitting in training data and spectrum bias (class imbalance) should be also considered by physicians, as they may have an impact on AI performance, and be avoided in evaluating the performance (63). It is logical that as the amount of data increases, the performance and accuracy of machine learning will also improve. However, it is difficult to develop a practical machine learning model, due to the paucity of hand-labeled data regarding confidentiality in private medical records (29). A method has been suggested to overcome this issue, which uses data reinforcement strategies (with artificially modified data) (64). Replacing the current ANN methods with more powerful computing capability of the neural networks could help minimize these problems (65). The primary purpose of training these neural networks is to enhance diagnosis and differentiate between features of hyperplastic and adenomatous polyps using thousands of medical polyp images from the database (66). Currently, recent studies based on recorded colonoscopy films or medical images have shown that polyp pathology with accuracy ranging from 70% to 96% can be predicted by this neural network model. On the other hand, by using negative predictive value techniques, this prediction increases to over 90% (37, 67-74). Effectiveness in real clinical practice does not mean that AI uses the accuracy of recognition or classification. Complex inspection can prove the actual benefit of clinical results, cost effectiveness beyond academic performance and satisfaction of physicians (13, 63). Furthermore, AI is not complete; although, AI is designed to replace human intelligence, “augmented intelligence” appears to emphasize the fact that AI is planned to improve or raise human intelligence (36). Despite the fact that in medical practice, AI aims to improve the work process with increasing accuracy and decrease the number of unintentional faults, imprecise data with incorrect performance in created models lead to incorrect grouping or diagnosis (18).

A crucial part of healthcare operation and function of medicine is the influence of AI on the communication between doctors and patients, which has not been evaluated. Hence, as soon as AI research begins to grow, the AI technique extension should be established (75). This AI platform accelerates the assumption of a ‘resect and discard’ strategy for diminutive colorectal polyps if accredited in designed clinical tests with patients during live procedures (71). In the future, a similar deep learning approach could facilitate the endoscopic examination by highlighting detection areas of possible adenomatous or serrated mucosa for accurate inspection (16). Moreover, the method described here is used for training programs that can distinguish between hyperplastic polyps and adenomatosis and has the potential to improve the detection of different types of endoscopic images that can resolve clinical problems such as identification of dysplasia in Barrett’s esophagus and detection of intestinal metaplasia and dysplasia in the gastric mucosa (76). Computer-assisted polyp detection is materializing with the rise of auto fluorescence and AI methods/deep learning which require no operator for each part of a computer-assisted polyp detection process (60, 76). In addition, deep learning is still in its nascent stage and further research is needed to better understand its value for widespread clinical implementation of optical polyp diagnosis (35, 71). Accordingly, the widespread use of this new tool is still limited in the diagnosis of polyps in medical imaging. Most recent researches are based on pre-recorded colonoscopy images, and only a few studies contain real-time images and polyp detection during colonoscopy in patients (36). There is also no set of focused randomized tests for definitive confirmation of this method as a suitable alternative to pathology- or endoscopist-based diagnosis (77). 

## Conclusion

The application of AI in gastroenterology has revealed varied errors in terms of statistical or image processing starting from the 1950s when it was initially introduced. AI and subcategories such as Machine Learning and Neural Networks have evolved dramatically in recent years and play a crucial role in diagnosis of diseases in the gastroenterology field. Moreover, AI will improve in different aspects of healthcare services even though it is still in its infancy and has some pitfalls. Accordingly, promising results from recent studies have shown the effectiveness of this method in accurate diagnosis of polyps and consequently unsupervised widespread clinical implementation is imminent in the near future.
